# Research on Emotion Analysis and Psychoanalysis Application With Convolutional Neural Network and Bidirectional Long Short-Term Memory

**DOI:** 10.3389/fpsyg.2022.852242

**Published:** 2022-06-30

**Authors:** Baitao Liu

**Affiliations:** School of Education Science, Nanyang Normal University, Nanyang, China

**Keywords:** emotion analysis, convolutional neural networks, bidirectional long short-term memory, sentiment classification, text sentiment recognition

## Abstract

This study mainly focuses on the emotion analysis method in the application of psychoanalysis based on sentiment recognition. The method is applied to the sentiment recognition module in the server, and the sentiment recognition function is effectively realized through the improved convolutional neural network and bidirectional long short-term memory (C-BiL) model. First, the implementation difficulties of the C-BiL model and specific sentiment classification design are described. Then, the specific design process of the C-BiL model is introduced, and the innovation of the C-BiL model is indicated. Finally, the experimental results of the models are compared and analyzed. Among the deep learning models, the accuracy of the C-BiL model designed in this study is relatively high irrespective of the binary classification, the three classification, or the five classification, with an average improvement of 2.47% in Diary data set, 2.16% in Weibo data set, and 2.08% in Fudan data set. Therefore, the C-BiL model designed in this study can not only successfully classify texts but also effectively improve the accuracy of text sentiment recognition.

## Introduction

In today’s society, because people’s current social environment is unique and unprecedented, in the face of the rapid development of society and increasing personal pursuit, young people’s outlook on life and values has also undergone great changes. The Institute of Psychology of the Chinese Academy of Sciences found that in today’s society, young people aged 20–30 years bear the highest psychological and mental pressure in all age groups. Under such pressure, more and more young people are out of balance, and their psychological problems are becoming increasingly serious.

College students, as an important group in society and the pillar of the country’s future development, are prone to psychological abnormalities when they gradually change from students to contact society due to various reasons such as enrollment, employment, environment, education, feelings, and value orientation. Most of them are in a state of mental sub-health or even have mental disorders or mental diseases, and some even mistakenly choose suicide, which have an irreparable negative impact on their families, schools, and society. Therefore, it is of great social significance to develop an application that can accurately identify the psychological state of young people, provide psychological help, and provide correct value orientation.

With the continuous development of artificial intelligence, machine learning, deep learning, and other technologies, natural language processing technology has also made great progress, including sentiment recognition of text, especially in dichotomies. The accuracy rate has been over 90%, and the accuracy rate of multi-classification has been improved with the continuous optimization of the neural network model. Therefore, the pertinence and efficiency of sentiment recognition in diary text can be further studied.

Sentiment recognition is also called sentiment analysis, and sentiment analysis for text is the focus of current research, involving the applications of natural language processing, machine learning (Yi et al., 2022), artificial intelligence, information retrieval, deep learning (Bao and Wang, 2022), and other fields. The main function of sentiment recognition is to enable computers to recognize complex human emotions. In the field of neural networks in deep learning, Kalchbrenner et al. (2014) proposed the DCNN model to improve semantic synthesis by studying the sentence structure. Santo Sand Gatti et al. used the convolutional neural network based on the sentence set and character set to carry out sentiment recognition of short texts, achieving high accuracy (dos Santos and Gattit, 2014). Severyn et al. expressed the feature of sentence meaning of text by training word vector and used the convolutional neural network to extract the deep-seated feature classifier (Severyn and Moschitti, 2015). Shi et al. (2016) proposed a discontinuous non-linear feature mapping classification model based on long and short memory neural networks. Yang et al. (2018) input the vector into the bidirectional LSTM layer and then input the result into the CNN for result fusion, which effectively improved the recognition accuracy. Different from the aforementioned research, this study uses the deep learning model combining the CNN and bidirectional LSTM and fully considers the original semantics and the sequential connection between the CNN and bidirectional LSTM, so as to improve the classification effect.

## Materials and Methods

This section describes the sentiment recognition method in the application of psychoanalysis based on sentiment recognition. In this study, an improved C-BiL model is proposed to effectively realize sentiment recognition. This section first describes the implementation difficulties of using the C-BiL model and specific sentiment classification design, then introduces the specific design process of the C-BiL model, and shows the innovation of the C-BiL model. Finally, the experimental results of the model are compared and analyzed.

### Design of the C-BiL Model

**FIGURE 1:**
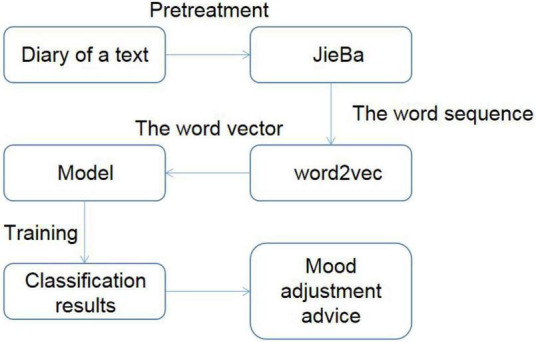
Realization process of the emotion recognition method.

This study is a psychological analysis application based on sentiment recognition. The main innovation of the function is that users write diaries on the Android client. Through certain methods, we can identify the emotional tendency of diaries so as to know the psychological state of users and give different psychological counseling suggestions according to the different psychological states of users. The difficulty lies in how to accurately identify the emotional tendency of the diary. The realization process of the whole sentiment recognition method is as follows: the user enters the diary text first. Diary text is a long text. Since there is no blank in Chinese sentences, pre-processing is required first. This method uses JieBa open-source tool to divide each sentence into words called word segments. By JieBa segmentation, each statement can be converted to a space-separated sequence of Chinese words. The preprocessed word sequence is input into the word2vec tool, and the word vector is trained in the data set specified in this article. Since the word vector cannot express emotional orientation, it is necessary to use a certain model for training so as to obtain text classification results that can express emotional orientation. According to the identified user emotions, different suggestions for mood adjustment should be given. The realization process of emotion recognition method is shown in Figure 1.

At the same time, due to the particularity of Chinese diary text, diary text will be full of users’ rich emotions, and it cannot express all users’ delicate emotions through simple “positive emotions,” “negative emotions,” and “neutral emotions.” Therefore, according to the professional knowledge of psychological science and combined with the mental health status of contemporary college students, this study designed five emotional results: “pleasure and relaxation,” “cool and calm,” “anxiety and confusion,” “sadness and self-blame,” and “pain and despair.”

“Pleasure and relaxation” mainly refers to the user is in a relatively relaxed emotional psychology. Diary content mainly expresses a happy mood or positive energy content, such as “I believe that as long as it is hard and happy, life is full and beautiful” and “let life be beautiful, and be positive and optimistic to meet every tomorrow.”

“Cool and calm” mainly indicates that the user is in a relatively objective and calm emotional psychology. The diary content mainly expresses a kind of calm, relatively flat emotion or normal narrative content, such as “for me, what I insist on will not be happy,” “let it be natural to deal with life and adapt to this change,” and “I met Wang Xiaoming on the road today.”

“Anxiety and confusion” mainly denotes that the user is in a more anxious and worried mood. The diary content mainly expresses the uneasiness of the unknown future or worries about some things, such as “Ideal, where are you?,” “I don’t know where I am going,” and “The course is so difficult, what should I do when the exam is coming.”

“Sadness and self-blame” mainly shows that the user is in a relatively sad emotional psychology. The diary mainly expresses the existence of a crying phenomenon or the content of chagrin about something, such as “I have always been strong and cried in public,” “I cried my heart and lungs in the middle of the night,” and “why do I hate myself so much.”

“Pain and despair” mainly conveys the user is very sad or close to crazy emotional psychology. The diary mainly expresses their own irrepressible sad mood or despair attitude toward a certain thing, such as “the world is a hell!,” “Are the living happier than the dead?,” and “I have no courage to continue living.”

In terms of suggestions on emotional adjustment, this article provides a variety of emotional adjustment methods and suggestions for the five different psychological emotions mentioned earlier. For example, for the “pleasure and relaxation” emotions, users are recommended to share happy things in life with friends or social networking sites to record every good thing in life. For “cool and calm” mood, it is recommended that users can use relatively calm mood for appropriate study or work, reasonable arrangement of time, and improve themselves. For “anxiety and confusion,” it is recommended that users find their family members or classmates and teachers to communicate with each other. Do not beat yourself up about it and get more hands-on experience, such as getting an internship or attending a school event. For the “sadness and self-blame” mood, users were recommended to take appropriate exercise, go to the playground to run or play ball to relieve the sadness and remorse mood, and calm the heart in the exercise. Finally, it is recommended that users call their closest family members or friends to have a chat with them, or go out for fun. They can also focus on their work and study and forget about other things, so as to divert their attention and relieve their severe emotions. The function of providing mood adjustment suggestions is realized in the emotion recognition module of the server in the following article.

#### Difficulties in Sentiment Recognition for Diary Text

1. The diary text has its particularity. Due to the variable length of the text, sometimes, it even varies greatly, leading to two difficulties: First, when the diary text is very short, for example, “I watched 007 today, it is not good.” In such a short context, how to decide whether 007 is a movie name or a number will have a certain impact on the final emotional judgment. Second, when the diary text is very long, it is likely to appear the whole diary before and after different emotional tendencies; for example, the beginning is still describing the sunny weather, but the end revealed the sad mood. Such long texts also interfere with sentiment recognition.
2. Different texts and different text classification methods have different feature extraction methods. A single model cannot extract the features uniformly. For example, the CNN only pays attention to the relevance of continuous words without fully considering the relevance of non-continuous words. Although the LSTM model has the correlation of long-distance words, it has no distinct feature extraction. As diary text describes human emotions, it is necessary to fully consider both feature extraction of the diary and emotional coherence or turning point of the full text in emotion recognition, so a single model cannot have a good recognition effect.
3. A separate CNN model or LSTM model is usually only used for binary and tertiary classification recognition. In this study, five categories of emotions are designed according to the different emotions that may be generated by users’ diary texts. Using a single model may have a higher accuracy in binary and tertiary classification, but it cannot improve the performance in quintic classification. Therefore, it is very important to design a targeted sentiment recognition model for diary text.

**FIGURE 2:**
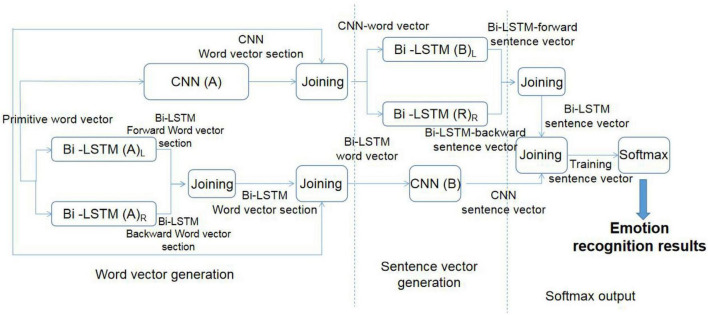
Structure of the C-BiL model.

In view of the aforementioned difficulties, the CNN achieves text sentiment recognition by extracting significant features of the measured data, while Bi-LSTM enhances long-distance text sentiment recognition by improving the generalization ability of the model and can take the semantics of the context into account. Therefore, the CNN and Bi-LSTM have their own advantages and emphases in emotional recognition of different types of texts. A new improved emotion recognition model, C-BiL model, was proposed. The model combines the advantages of the CNN and LSTM algorithm to achieve more accurate emotion recognition. The structure of the C-BiL model is shown in Figure 2.

The whole C-BiL model is divided into three layers. Among them, CNN(A) and CNN(B); Bi-LSTM(A) and Bi-LSTM(B), respectively, represent two sets of parameter rules of the CNN and Bi-LSTM algorithm. The design idea of the C-BiL model is given as follows:

#### The First Layer: Generation of the CNN and Bi-LSTM Word Vector

The original word vector is trained through the CNN(A) to get the corresponding CNN word vector segment.

The positive order of the original word vector is input into Bi-LSTM(A)_*L*_, and the corresponding Bi-LSTM forward word vector segment is trained. The original word vector is input into Bi-LSTM(B)_*R*_ in a reverse order, and the corresponding Bi-LSTM backward word vector segment is trained. The Bi-LSTM forward word vector segment and Bi-LSTM backward word vector segment are spliced to obtain the Bi-LSTM word vector segment.

The generated CNN word vector segment and Bi-LSTM word vector segment are spliced with the original word vector to generate the CNN word vector and Bi-LSTM word vector, respectively.

#### The Second Layer: CNN and Bi-LSTM Sentence Vector Generation

The CNN word vector is input into Bi-LSTM(B)_*L*_, and Bi-LSTM forward sentence vector is obtained by training. The CNN word vector is input in a reverse order into Bi-LSTM(B)_*R*_, and the Bi-LSTM backward sentence vector is obtained by training. The Bi-LSTM forward sentence vector is spliced with the Bi-LSTM backward sentence vector to obtain the Bi-LSTM sentence vector.

Bi-LSTM word vector is input into the CNN(B), and The CNN sentence vector is obtained by training.

#### The Third Layer: Softmax Output

First, the Bi-LSTM sentence vector and CNN sentence vector are spliced to get the training sentence vector. The training sentence vector is input into Softmax layer to get the final emotion recognition result.

Based on the aforementioned functional requirements of emotion recognition, the C-BiL model proposed in this article can not only accurately identify the five emotions in diary text but also effectively improve the accuracy of recognition and ensure the realization of application functions.

#### Innovations of the C-BiL Model

1. In the field of text sentiment recognition, many models are based on the CNN only, some models are based on the LSTM only, and some improved models are based on the Bi-LSTM. Due to the particularity of diary text, it is required to fully extract text features and fully combine contextual semantics. Therefore, by combining the advantages of the CNN in capturing local features and LSTM in capturing timing features and considering context semantics, this study improved LSTM to Bi-LSTM, so as to obtain a C-BiL model with better performance.
2. At present, there are many models that combine the CNN and LSTM algorithm. In some models, vectors are input into the CNN and Bi-LSTM, respectively, for encoding, and relevant features are extracted. Finally, the two are fused at the full connection layer for classification network. In some models, vectors are input into the CNN to extract relevant features, and the results are input into the Bi-LSTM layer after passing through the convolutional layer to finally classify. In some models, vectors are input into the Bi-LSTM layer and then the results are input into the CNN (Shi et al., 2016). Some models simultaneously verified three combinations of the CNN and Bi-LSTM: the CNN followed by LSTM, LSTM followed by the CNN, and the CNN in parallel with LSTM. In this study, the C-BiL model generates CNN word vector segments and Bi-LSTM word vector segments, first splicing with the original word vector to ensure that the original word meaning is not lost in the training process. Then, Bi-LSTM and CNN are fused, respectively, to generate Bi-LSTM sentence vectors and CNN sentence vectors. Finally, the two sentence vectors are joined together and input into Softmax to obtain the final classification result. This model not only retains the original semantics to the maximum extent but also fully integrates the CNN and Bi-LSTM. The CNN is followed by Bi-LSTM, and Bi-LSTM is followed by the CNN. The final result also fully integrates the extracted features of the two (Graves et al., 2005; Collobert et al., 2011; Cho et al., 2014; Tai and Socher, 2015; Er et al., 2016; Brachmann et al., 2017; Huang et al., 2017; Jebbara and Cimiano, 2017; Wigington et al., 2017; Yenter and Verma, 2017; Huebner and Willits, 2018).
3. The general models are binary or tripartite experiments on specified data sets. Due to the particularity of diary text, the emotions contained in the text are relatively complex, and binary or tertiary classification cannot fully and accurately express users’ emotions. Therefore, this model designs five classification results.

In summary, the C-BiL model has better performance.

##### CNN Algorithm Design

The CNN is generally used in computer vision, especially in image processing. On the one hand, since people do not pay too much attention to global pixels when they focus on an image, more local features can be extracted through convolution. This approach is more consistent with the daily image processing. Since each individual neuron only needs to perceive local features, global information can be obtained by integrating several local features at a higher level, which eliminates the perception of global images by each neuron, thus greatly improving efficiency. On the other hand, the CNN has the feature of weight sharing, and only extracting the maximum features of sub-intervals while ignoring other features can greatly reduce the parameter scale of the model. Finally, because the parameters of a convolutional kernel are fixed, the extracted features will be relatively simple, but the CNN can have multiple convolutional kernels, so that things can be analyzed from more perspectives, making the final results more objective and avoiding bias.

Different from images with pixels, in terms of natural language processing, the object is usually a document or sentence, while the input of the CNN needs to be a matrix. Therefore, the word2vec method is used in this model to train every word in every sentence in the diary text into the word vector. Each behavior has a word vector, and there are as many lines of word vectors as there are words. Therefore, the diary text can be converted into a word vector matrix as the input of the CNN algorithm.

The input layer, convolution layer, pooling layer, and full connection layer constitute the network model involved in this study. In the input layer, the text input word vector features are connected in series, and the vector features are extracted from the convolution layer and the pooling layer. The final text classification result is obtained by the Softmax function at the full connection layer. The specific implementation process of the model is described as follows:

Input layer: The input text is converted from words to word vectors to construct feature matrix: *x*_1:*L*_ = *x*_1_⊕*x*_2_⊕⋯⊕*x*_*L*_,

Where any word vector is represented by x_i_, representing the word vector corresponding to the i-th word in a sentence, and the number of words in a sentence is represented by L. In this model, the dimension of the trained word vector is 300, and the distance is calculated by cosine, where the symbol “⊕” represents the series operation, which means that L 1*300 word vectors can be connected in series into an L*300 eigenmatrix. To ensure that the number of words in each sentence is L, 0 is added when the text length is less than L words, and the text is truncated with length L when the text length is greater than L words.

Convolution layer: It performs convolution operations on input features.


(1) $$c_{i,j}=f\,\sum_{m=0}^{M}\sum_{n=0}^{N}w_{m,n}\,x_{i+m,j+n}+w_{b}$$

The formula is simplified to obtain:


(2) $${\text{c}}_{\text{i}}=\text{f}\,\left(\text{w}\cdot {\text{x}}_{i:i+h-1}+\text{b}\right)$$

where the element of the i-th row and j-th column of the input feature is represented by x_i,j_, the weight of the m-th row and n-th column in the convolution kernel is represented by w_m,n_, the bias term of the convolution kernel is represented by w_b_, and the element of the i-th row and j-th column in the feature graph of the convolution result is represented by c_i,j_, f represents the activation function, and the ReLU function is used in this model. The value of each element in the output matrix is changed to 0 for positions less than 0.

After the convolution operation in the convolution layer, the scale of the eigenmatrix decreases as follows:


(3) $$W_{0}=\left(W_{1}-F\right)/+1$$

(4) $$H_{0}=\left(H_{1}-F\right)/+1$$

where *W_0_* is the width of the feature graph after convolution; *W_1_* is the width of the feature matrix before convolution; *F* and S, respectively, represent the width and the step of the convolution kernel, where S = 1; *H_0_* is the height of the feature graph after convolution; and *H_1_* is the height of the feature graph before convolution.

Pooling layer: The maximum pooling method of Max Pooling is adopted in this model, where:


(5) $$h_{i}=m\,a\,x\,p\,o\,o\,l\,i\,n\,g\,\left\{c_{i\,1},c_{i\,2},\cdots ,c_{i\,n-1}\right\}$$

In this formula, *h_i_* represents the output of the pooling layer, and the sample value after sampling is the maximum value of the m*n sample selected in the maximum pooling layer. In this sampling method, unimportant samples in the feature graph can be ignored to reduce the number of parameters.

Full connection layer: The full connection layer is the full connection calculation of *h_i_*:


(6) $$o_{j}=s\,i\,g\,m\,o\,i\,d\,\left(\sum_{i}w_{j,i}\cdot h_{i}+w_{j,b}\right)$$

*o_j_* is the output value of the full connection layer, and the output result is classified into the Softmax function to get the final result.

So far, the CNN algorithm in the C-BiL model has been designed.

##### Bi-LSTM Neural Network Algorithm Design

LSTM is a variant of the RNN model, which can solve the long-term dependence problem of the RNN. The key of LSTM is the state of the cell, and the addition or deletion of information is realized through the gate structure. The gate structure implements selective passage of information through a sigmoid neural layer and point-by-point multiplication operation. LSTM has three gates, namely, the input gate, the forgetting gate, and the output gate.

Input gate: This gate shows how much new information is allowed through the current cell state. The sigmoid layer decides what information needs to be updated, and the tanh layer generates a content vector $\tilde{C_{t}}$ for alternate updates. See formulas 8, 9:


(7) $$i_{t}=\sigma \,\left(W_{i}\cdot \left[h_{t-1},x_{t}\right]+b_{i}\right)$$

(8) $$\tilde{C_{t}}=t\,a\,n\,h\,\left(W_{c}\cdot \left[h_{t-1},x_{t}\right]+b_{c}\right)$$

Combining the previous two steps, the cell state can be updated, in this case to G. The cT-I state is multiplied by f to get the information to be discarded and forgotten. The new candidate value is I, Ct, which can be changed by the degree of updating each state, as shown in Formula 10:

Combining the previous two steps, the cell state can be updated, where *C*_*t–1*_ is updated to *C_t_*. The *C*_*t–1*_ state is multiplied by *f_t_* to get the information to be discarded and forgotten. The new candidate value is $i_{t}\cdot \tilde{C_{t}}$, which can be changed by the degree of updating each state, as shown in formula 10:


(9) $$C_{t}=f_{t}\cdot C_{t-1}+i_{t}\cdot \tilde{C_{t}}$$

Output gate: It determines the output value of the current cell state. The sigmoid layer first determines which part of the output cell state, and after tanh processing, the result is between [−1,1], which is then multiplied by the output of the sigmoid layer to obtain the final output result, as shown in formulas 11, 12:


(10) $$o_{t}=\sigma \,\left(W_{0}\cdot \left[h_{t-1},x_{t}\right]+b_{0}\right)$$

(11) $$h_{t}=o_{t}\cdot t\,a\,n\,h\,\left(C_{t}\right)$$

It can be seen from the derivation of the formula of LSTM that the current cell state *C_t_*is jointly determined by the previous cell state and the current cell information. However, when faced with a long diary text consisting of multiple sentences, context information has to be considered. The standard LSTM model only considers the time sequence information and ignores the subsequent information, so Bi-LSTM model is adopted in this model to improve the performance better.

Bi-LSTM consists of forward LSTM and reverse LSTM, which are independent of each other and have the same structure. The forward LSTM uses forward contextual semantic information as input, and the reverse LSTM uses reverse contextual semantic information as input, that is, the preceding information can be obtained through the forward LSTM, and the following information can be obtained through the reverse LSTM. Bi-LSTM ensures that the information before and after the diary text can be factored in to achieve higher performance. In this model, Bi-LSTM_*L*_ processes the forward input sequence and Bi-LSTM_*R*_ processes the reverse input sequence, and the two share a set of parameters during model training.

The final output *h_t_* of Bi-LSTM can be obtained by splicing forward output $\underset{h_{t}}{⟶}$ and backward output $\underset{h_{t}}{⟵}$:


(12) $$h_{t}=\underset{h_{t}}{⟶}⊕\underset{h_{t}}{⟵}$$

So far, the Bi-LSTM algorithm in the C-BiL model has been designed. Next, the whole C-BiL model is designed.

##### CNN and Bi-LSTM Word Vector Generation

In this model, *x*_*i*_ ∈ ^*Rd*^ represents the word vector features of each input word and d represents the dimension of word vector. Thus, x ∈ ^RL×d^ represents the input feature matrix of N words. When the input is a sentence, it can be expressed as follows:


(13) $$X_{1:N}=x_{1}⊕x_{2}⊕\cdots ⊕x_{N}$$

where ⊕ indicates the splicing operation. The maximum length is set as N. When the length is insufficient, the eigenmatrix is supplemented with 0, and when the length exceeds N, the eigenmatrix is truncated. Therefore, the length combination of any paragraph of word vector can be expressed as follows:


(14) $$X_{j:j+m}=x_{j}⊕x_{j+1}⊕\cdots ⊕x_{j+m}$$

Word vector *X*_*1:N*_ is used as the input vector of CNN(A) and Bi-LSTM (A) algorithms.

###### Generation of CNN Word Vector

The CNN(A) algorithm adopts K eigenmatrices of the convolution check input for convolution operation, where c is the j-th feature generated by the k-th convolution:

The CNN(A) algorithm adopts K convolution kernels to carry out the convolution operation on the input eigenmatrix. $c_{j}^{k}$ is the j-th feature generated by the k-th convolution:


(15) $$c_{j}^{k}=f_{A}\,\left(W_{A}⊗X_{j:j+h-1}+b_{A}\right)$$

⊗ stands for the convolution operation, *f_A_* is the activation function of CNN(A), and the ReLU function is selected as the activation function in this system. The size of the sliding window is denoted as *h_A_*, and the offset value is denoted as *b_A_*. *X*_*j:j+h–1*_ represents the local eigenmatrix consisting of rows j-th through j+h–1-th. Therefore, the feature vector $c_{j}^{k}$ obtained after the convolution operation is as follows:


(16) $$c_{j}^{k}=\left[c_{j\,1},c_{j\,2},\cdots \,c_{j\,N-h+1}\right]$$

where j represents the serial number of the convolution kernel. An eigenvector $c_{j}^{k}$ can be obtained through the convolution operation of multiple convolution kernels. Splicing N $c_{j}^{k}$ vectors to obtain the output CNN word vector segment *C*_*A_1_:N*_:


(17) $$C_{A_{1:N}}=C_{1:N}^{1}⊕C_{1:N}^{2}⊕\cdots ⊕C_{1:N}^{K}$$

Let the original word vector *X*_*1:N*_ and CNN word vector segment *C*_*A_1_:N*_ merge into *A^c*_*1:N*_ :


(18) $$A_{1:N}^{C}=X_{1:N}⊕C_{A_{1:N}}$$

Splicing N *A_C_* into final output CNN word vector $A_{t}^{c}$:


(19) $$A_{t}^{c}=A_{1}^{C}⊕A_{2}^{C}⊕\cdots ⊕A_{N}^{C}$$

###### Bi-LSTM Word Vector Generation

First, the forward word vector of Bi-LSTM(A)_*L*_ is calculated. Taking vector X as the input vector of the Bi-LSTM(A)_*L*_ algorithm, the corresponding hidden status update of the j-th word vector is as follows:


(20) $$\underset{f_{J}^{A}}{⟶}=\sigma \,\left(W_{f}^{A}\cdot \left[\underset{h_{J-1}}{⟶},\underset{X_{1:N}}{⟶}\right]+b_{f}^{A}\right)$$

(21) $$\underset{l_{J}^{A}}{⟶}=\sigma \,\left(W_{i}^{A}\cdot \left[\underset{h_{J-1}}{⟶},\underset{X_{1:N}}{⟶}\right]+b_{i}^{A}\right)$$

(22) $$\underset{{\tilde{C}}_{J}^{A}}{⟶}=t\,a\,n\,h\,\left(W_{C}^{A}\cdot \left[\underset{h_{J-1}}{⟶},\underset{X_{1:N}}{⟶}\right]+b_{C}^{A}\right)$$

(23) $$\underset{C_{J}^{A}}{⟶}=\underset{f_{J}^{A}}{⟶}\cdot \underset{C_{J-1}^{A}}{+}⟶\underset{l_{J}^{A}}{⟶}\cdot \underset{{\tilde{C}}_{J}^{A}}{⟶}$$

(24) $$\underset{o_{J}^{A}}{⟶}=\sigma \,\left(W_{j}^{A}\cdot \left[\underset{h_{J-1}}{⟶},\underset{X_{1:N}}{⟶}\right]+b_{o}^{A}\right)$$

(25) $$\underset{h_{J}^{A}}{⟶}=\underset{o_{J}^{A}}{⟶}\cdot t\,a\,n\,h\,\left(\underset{C_{J}^{A}}{⟶}\right)$$

where $\underset{l_{J}^{A}}{⟶}$ represents the input gate, $\underset{f_{J}^{A}}{⟶}$ represents the forget gate, $\underset{o_{J}^{A}}{⟶}$ represents the output gate, $\underset{{\tilde{C}}_{J}^{A}}{⟶}$ represents the candidate value of the cell state, $\underset{C_{J}^{A}}{⟶}$ represents the cell state of the updated j-th word vector, and $\underset{h_{J}^{A}}{⟶}$ represents the value of the hidden state of the j-th word vector.

The output vector of each word is $\underset{h_{J}^{A}}{⟶}$, and the output of N words is spliced to obtain the forward word vector segment $\underset{H_{A_{1:N}}}{⟶}$ of Bi-LSTM(A)_*L*_:


(26) $$\underset{H_{A_{1:N}}}{⟶}=\underset{h_{1}^{A}}{⟶}⊕\underset{h_{2}^{A}}{⟶}⊕\cdots ⊕\underset{h_{N}^{A}}{⟶}$$

Let the original word vector $\underset{X_{1}}{⟶}$ and the forward word vector segment $\underset{H_{A_{1:N}}}{⟶}$ of Bi-LSTM(A)_*L*_ merge into $\underset{A_{1:N}^{L}}{⟶}$:


(27) $$\underset{A_{1:N}^{L}}{⟶}=\underset{X_{1:N}}{⟶}⊕\underset{H_{A_{1:N}}}{⟶}$$

Splicing N $\underset{A_{1:N}^{L}}{⟶}$ into the forward word vector $\underset{A_{t}^{L}}{⟶}$ of the final output Bi-LSTM(A)_*L*_:


(28) $$\underset{A_{t}^{L}}{⟶}=\underset{A_{1}^{L}}{⟶}⊕\underset{A_{2}^{L}}{⟶}⊕\cdots ⊕\underset{A_{N}^{L}}{⟶}$$

Similarly, taking the reverse vector $\underset{X_{1:N}}{⟵}$ as the input vector of Bi-LSTM(A)_*R*_ algorithm, the backward word vector $\underset{A_{t}^{L}}{⟵}$ of Bi-LSTM(A)_*R*_ algorithm can be obtained:


(29) $$\underset{A_{t}^{L}}{⟵}=\underset{A_{1}^{L}}{⟵}⊕\underset{A_{2}^{L}}{⟵}⊕\cdots ⊕\underset{A_{N}^{L}}{⟵}$$

Splicing forward word vector $\underset{A_{t}^{L}}{⟶}$ and backward word vector $\underset{A_{t}^{L}}{⟵}$ to obtain the final Bi-LSTM word vector $A_{t}^{L}$:


(30) $$A_{t}^{L}=\underset{A_{t}^{L}}{⟶}⊕\underset{A_{t}^{L}}{⟵}$$

So far, CNN word vector $A_{t}^{c}$ and Bi-LSTM word vector $A_{t}^{L}$ are obtained through CNN(A) and Bi-LSTM(A).

##### CNN and Bi-LSTM Sentence Vector Generation

###### Bi-LSTM Sentence Vector Generation

First, the forward sentence vector of Bi-LSTM(B)_*L*_ is calculated. The forward word vector $\underset{A_{t}^{c}}{⟶}$ of the CNN is taken as the input vector of the Bi-LSTM(B)_*L*_ algorithm, and the corresponding hidden status update of the j-th word is as follows:


(31) $$\underset{f_{J}^{B}}{⟶}=\sigma \,\left(W_{f}^{B}\cdot \left[\underset{h_{J-1}}{⟶},\underset{A_{t}^{c}}{⟶}\right]+b_{f}^{B}\right)$$

(32) $$\underset{l_{J}^{B}}{⟶}=\sigma \,\left(W_{i}^{B}\cdot \left[\underset{h_{J-1}}{⟶},\underset{A_{t}^{c}}{⟶}\right]+b_{i}^{B}\right)$$

(33) $$\underset{{\tilde{C}}_{J}^{B}}{⟶}=t\,a\,n\,h\,\left(W_{C}^{B}\cdot \left[\underset{h_{J-1}}{⟶},\underset{A_{t}^{c}}{⟶}\right]+b_{C}^{B}\right)$$

(34) $$\underset{C_{J}^{B}}{⟶}=\underset{f_{J}^{B}}{⟶}\cdot \underset{C_{J-1}^{B}}{+}⟶\underset{l_{J}^{B}}{⟶}\cdot \underset{{\tilde{C}}_{J}^{B}}{⟶}$$

(35) $$\underset{o_{J}^{B}}{⟶}=\sigma \,\left(W_{j}^{B}\cdot \left[\underset{h_{J-1}}{⟶},\underset{A_{t}^{c}}{⟶}\right]+b_{o}^{B}\right)$$

(36) $$\underset{h_{J}^{B}}{⟶}=\underset{o_{J}^{B}}{⟶}\cdot t\,a\,n\,h\,\left(\underset{C_{J}^{B}}{⟶}\right)$$

where the input gate, forget gate, and output gate are denoted as $\underset{l_{J}^{B}}{⟶}$, $\underset{f_{J}^{B}}{⟶}$, and $\underset{o_{J}^{B}}{⟶}$, respectively, and the candidate value of cell state is denoted as $\underset{{\tilde{C}}_{J}^{B}}{⟶}$, the cell state of the updated j-th word vector is denoted as $\underset{C_{J}^{B}}{⟶}$, and the hidden state value of the i-th word vector is denoted as $\underset{h_{J}^{B}}{⟶}$.

The output vector of each word is $\underset{h_{J}^{B}}{⟶}$, and the output of N words is spliced to obtain Bi-LSTM forward sentence vector $\underset{H_{t}^{L}}{⟶}$:


(37) $$\underset{H_{t}^{L}}{⟶}=\underset{h_{1}^{B}}{⟶}⊕\underset{h_{2}^{B}}{⟶}⊕\cdots ⊕\underset{h_{2}^{B}}{⟶}$$

Similarly, the backward word vector $\underset{A_{t}^{L}}{⟵}$ of the CNN is taken as the input vector of the Bi-LSTM(B)_*R*_ algorithm, and the backward sentence vector $\underset{H_{t}^{L}}{⟵}$ of Bi-LSTM(B)_*L*_:


(38) $$\underset{H_{t}^{L}}{⟵}=\underset{h_{1}^{B}}{⟵}⊕\underset{h_{2}^{B}}{⟵}⊕\cdots ⊕\underset{h_{N}^{B}}{⟵}$$

By splicing forward sentence vector $\underset{{\text{A}}_{\text{t}}^{\text{L}}}{⟶}$ and backward sentence vector $\underset{{\text{A}}_{\text{t}}^{\text{L}}}{⟵}$, the final Bi-LSTM sentence vector ${\text{H}}_{\text{t}}^{\text{L}}$ is obtained:


(39) $$H_{t}^{L}=\underset{H_{t}^{L}}{⟶}⊕\underset{H_{t}^{L}}{⟵}$$

###### CNN Sentence Vector Generation

Bi-LSTM word vector $A_{t}^{L}$ is taken as input word vector of the CNN(B) algorithm. The CNN(B) algorithm adopts K convolution kernels to carry out convolution operation on the input eigenmatrix. Where $c_{j}^{k^{\prime }}$is the j-th feature generated by the k-th convolution:


(40) $$c_{j}^{k^{\prime }}=f_{B}\,\left(W_{B}⊗A_{j:j+h-1}^{L}+b_{B}\right)$$

⊗ stands for convolution operation, *f_B_* is the activation function of CNN(B), and the ReLU function is selected as the activation function in this system. The size of the sliding window is denoted as *h_B_*, and the offset value is denoted as *b_B_*. *A^L*_*j:j+h–1*_ represents the local eigenmatrix consisting of rows j-th through j+h-1-th. Therefore, the feature vector $c_{j}^{k^{\prime }}$ obtained after the convolution operation is as follows:


(41) $$c_{j}^{k^{\prime }}=\left[A_{j\,1}^{L},A_{j\,2}^{L},\cdots ,A_{j\,N-h+1}^{L}\right]$$

where j is the j-th convolution kernel. An eigenvector $c_{j}^{k^{\prime }}$ can be obtained through the convolution operation of multiple convolution kernels. Splicing N $c_{j}^{k^{\prime }}$ vectors to get output *C*_*B_1_:N*_:


(42) $$C_{B_{1:N}}=C_{1:N}^{1}⊕C_{1:N}^{2}⊕\cdots ⊕C_{1:N}^{K}$$

*C*_*B_1_:N*_ is input into the pooling layer to carry out the maximum pooling operation and obtain the CNN sentence vector $H_{t}^{C}$:


(43) $$H_{t}^{C}=m\,a\,x\,p\,o\,o\,l\,i\,n\,g\,\left\{C_{B_{1}},C_{B_{2}},\cdots ,C_{B_{N}}\right\}$$

So far, the CNN sentence vector $H_{t}^{C}$ and BI-LSTM sentence vector $H_{t}^{L}$ are obtained through CNN(B) and Bi-LSTM(B).

#### Output From the Softmax Layer

Softmax is widely used in machine learning and deep learning, especially in multi-classification scenarios. The final output unit of the classifier requires the Softmax function for numerical processing (Li et al., 2020). The Softmax functions are defined as follows:


(44) $$S_{i}=\frac{e^{V_{i}}}{\sum_{i}^{C}e^{V_{i}}}$$

where *V_i_* is the output of the classifier’s pre-output unit, i represents the category index, the total number of categories is C, and *S_i_* represents the ratio of the index of the current element to the index sum of all elements. Softmax maps some inputs to real numbers between [0,1], and the normalization guarantees that the sum is 1, and the sum of the probabilities of the multiple categories is exactly 1. Therefore, Softmax can convert the output values of multiple categories into relative probabilities.

In the C-BiL algorithm model, since CNN sentence vectors and Bi-LSTM sentence vectors have been obtained, the sentence vectors of the two parts are spliced to obtain the training sentence vector O′:


(45) O=′HtC⊕HtL

O′ is input into the Softmax layer for classification:


(46) $$\text{y}=\text{softmax}\,\left({\text{W}}_{\text{s}}\,O^{\prime }+{\text{b}}_{\text{s}}\right)$$

Here, W_s_ ∈ ^Rd^ and b_s_ are parameters of the Softmax layer, and b_s_ is the number of classifier classifications.

So far, the final classification result y is obtained, and the whole C-BiL model can complete the function of sentiment recognition.

## Experiment and Analysis

This section conducts experiments on the C-BiL model to obtain the accuracy of the model.

### Data Set

This model uses three data sets to conduct experiments, namely, Weibo, Fudan, and Diary.

(1) Weibo (Data_1): The data of Sina Weibo is manually collected and labeled. The sample totals 3 million. The experiment randomly selects 40,000 positive emotion data, 50,000 negative emotion data, and 10,000 neutral emotion data as the data set.
(2) Fudan (Data_2): The Fudan University Article Classification Collection is a collection of Chinese articles with nearly 11,000 entries in 20 categories, including art, education, and energy.
(3) Diary (Data_3): In order to train the model, diary texts from the online side about mental health are captured by hand.

Table 1 provides details for each data set, including the number of data set classes (C), the number of training sets (Train)/validation sets (Dev)/test sets (Test), and the average text length (Len).

**TABLE 1:** Data set information.

Dataset	C	Train/Dev/Test	Len
Weibo	3	80000/10000/10000	568
Fudan	20	8823/981/9832	2981
Diary	5	1900/200/200	540

### Model Training and Result Analysis

The word vector generator is designed to generate a distributed representation of each word. A common approach to improving performance in the absence of large supervised training sets is to obtain word vector initializations from unsupervised neural language models. In this algorithm model, word vectors are trained in three datasets of Weibo, Fudan, and Diary using the public word2vec tool. The vector has a dimension of 300 and is trained using a continuous skip-gram architecture. It is worth noting that since there is no white space in Chinese sentences, and pre-processing work is required to divide each sentence into word segments with Chinese white space. Here, JieBa open-source tools are used to achieve this goal. By JieBa segmentation, each statement can be converted to a space-separated sequence of Chinese words.

In order to test the accuracy of the C-BiL model, this study conducted comparative experiments with the following four models:

(1) SFCNN (Kim et al., 2019): The convolutional neural network is used for text feature learning after multi-channel semantic synthesis of the word vector.
(2) Bi-LSTM (Zou et al., 2021): Only the Bi-LSTM neural network is used for text feature learning.
(3) CNN-BiLSTM (Zhang et al., 2020): The CNN and Bi-LSTM are input, respectively, for encoding; relevant features are extracted, and finally classified them by fusion in the full connection layer.
(4) BiLSTM-CNN (Zhu et al., 2019): The vector is input into Bi-LSTM and then the result is input into the CNN for final classification.

As the model proposed in this article, the C-BiL model is shown in Table 2 for parameter configuration:

**TABLE 2:** Parameters settings for the C-BiL model.

Adjustable parameter	The value selected
Word vector dimension	300
W_A_, b_A_, ${\text{W}}_{\text{f}}^{\text{A}}$, ${\text{b}}_{\text{f}}^{\text{A}}$, ${\text{W}}_{\text{i}}^{\text{A}}$, ${\text{b}}_{\text{i}}^{\text{A}}$, ${\text{W}}_{\text{C}}^{\text{A}}$, ${\text{b}}_{\text{C}}^{\text{A}}$, ${\text{W}}_{\text{j}}^{\text{A}}$, ${\text{b}}_{\text{o}}^{\text{A}}$	Random is uniformly distributed initially (−0.1,0.1)
W_B_, b_B_, ${\text{W}}_{\text{f}}^{\text{B}}$, ${\text{b}}_{\text{f}}^{\text{B}}$, ${\text{W}}_{\text{i}}^{\text{B}}$, ${\text{b}}_{\text{i}}^{\text{B}}$, ${\text{W}}_{\text{C}}^{\text{B}}$, ${\text{b}}_{\text{C}}^{\text{B}}$, ${\text{W}}_{\text{j}}^{\text{B}}$, ${\text{b}}_{\text{o}}^{\text{B}}$	Random is uniformly distributed initially (−0.1,0.1)
CNN(A) convolution kernel function	ReLu
CNN(B) convolution kernel function	ReLu
CNN(A) filter sliding window size	300*2
CNN(B) filter sliding window size	600*2
CNN(A) number of filters	300
CNN(B) number of filters	300
Random update parameter ratio	0.5
Number of model training iterations	50
Number of LSTM(A)’ s layers	1
Number of LSTM(b)’ s layers	1
Number of LSTM(A)’ s hidden units	300
Number of LSTM(B)’s hidden units	300

Tables 3, 4, 5 represent the experiment classification result with two, three, and five types, respectively.

**TABLE 3:** Accuracy of dichotomies.

Model comparison	Weibo	Fudan	Diary
SFCNN (Benchmark model_1)	93.79%	93.04%	94.16%
Bi-LSTM (Benchmark model_2)	94.83%	94.60%	94.97%
CNN-BiLSTM (Benchmark model_3)	94.96%	94.74%	95.04%
BiLSTM-CNN (Benchmark model_4)	94.89%	94.83%	95.19%
C-BiL (The proposed model)	95.66%	95.77%	96.12%

**TABLE 4:** Accuracy rate under three categories.

Model comparison	Weibo	Fudan	Diary
SFCNN (Benchmark model_1)	82.34%	81.93%	83.51%
Bi-LSTM (Benchmark model_2)	82.56%	82.07%	83.73%
CNN-BiLSTM (Benchmark model_3)	82.16%	81.57%	83.98%
BiLSTM-CNN (Benchmark model_4)	83.63%	82.30%	84.07%
C-BiL (The proposed model)	83.94%	83.64%	84.89%

**TABLE 5:** Accuracy rate under five categories.

Model comparison	Fudan	Diary
SFCNN (Benchmark model_1)	54.90%	56.07%
Bi-LSTM (Benchmark model_2)	54.72%	55.93%
CNN-BiLSTM (Benchmark model_3)	54.96%	56.29%
BiLSTM-CNN (Benchmark model_4)	55.17%	56.34%
C-BiL (The proposed model)	56.45%	57.56%

As shown in Tables 3, 4, 5, this study introduces three data sets and uses four models to classify data, among which Weibo is a three-category data set, Fudan is a multi-category data set, and Diary is a five-category data set. On the whole, since the application is for sentiment recognition of diary text, the manual-labeled Diary data set has a higher accuracy rate than Weibo and Fudan data sets (0.69% higher on average) because the training content is closer to the actual demand.

In the dichotomous experiment, the accuracy of the proposed method in Diary data set is 0.93% better than Benchmark model_4, 1.08% better than Benchmark model_3, 1.05% better than Benchmark model_2, and 1.86% better than Benchmark model_1. In the data set of Weibo, the accuracy is 0.77% better than Benchmark model_4, 0.7% better than Benchmark model_3, 0.63% better than Benchmark model_2 and 1.67% better than Benchmark model_1. In The Fudan data set, the accuracy is 0.94% better than Benchmark model_4, 1.03% better than Benchmark model_3, 0.77% better than Benchmark model_2 and 2.33% better than Benchmark model_1.

In the three classification experiments, the accuracy of the proposed method in Diary data set is 0.82% better than Benchmark model_4, 0.91% better than Benchmark model_3, 0.86% better than Benchmark model_2, and 1.08% better than Benchmark model_1; In the data set of Weibo, the accuracy is 0.31% better than Benchmark model_4, 1.78% better than Benchmark model_3, 1.38% better than Benchmark model_2 model, and 1.60% better than Benchmark model_1 model. In The Fudan data set, the accuracy is 1.34% better than Benchmark model_4, 2.07% better than Benchmark model_3, 1.37% better than Benchmark model_2, and 1.51% better than Benchmark model_1.

In the five-category experiment, the accuracy of the proposed method in Diary data set is 1.22% better than Benchmark model_4, 1.27% better than Benchmark model_3, 1.23% better than Benchmark model_2, and 1.09% better than Benchmark model_1. In The Fudan dataset, the accuracy is 1.28% better than Benchmark model_4, 1.49% better than Benchmark model_3, 1.53% better than Benchmark model_2, and 1.35% better than Benchmark model_1.

In conclusion, among the deep learning models, the accuracy of the C-BiL model designed in this study is relatively high irrespective of the binary classification, the three classification, or the five classification, with an average improvement of 2.47% in the Diary data set, 2.16% in the Weibo data set, and 2.08% in the Fudan data set.

Therefore, the C-BiL model designed in this study can not only successfully classify texts but also effectively improve the accuracy of text sentiment recognition.

## Conclusion

This article mainly introduces the design and implementation of a psychological analysis application based on emotion recognition algorithm, which is mainly aimed at college students. With the occurrence and reports of adverse events on campus one after another, college students’ mental health problems have gradually become the focus of society. These problems may be general psychological problems of growth or may be psychological disorders caused by various social or environmental reasons. Facing up to and solving these problems have become the task that society, colleges, and families must face. The psychological analysis application based on sentiment recognition designed in this study can meet the established requirements of initial application, and the C-BiL model is proposed by improving the convolutional neural network model and the Bi-LSTM neural network model, which effectively improve the accuracy of emotion recognition. However, there are still areas for further improvement in the algorithm design of this study, for example, in the Bi-LSTM algorithm, attention mechanism can be introduced to assign different weights to different parts of speech of diary text, such as adjectives, nouns, and adverbs so that accuracy can be improved more effectively in emotion recognition. The first and last sentences of the diary can also be weighted to improve the recognition efficiency.

## Data Availability Statement

The original contributions presented in the study are included in the article/supplementary material, further inquiries can be directed to the corresponding author.

## Author Contributions

The author confirms being the sole contributor of this work and has approved it for publication.

## Conflict of Interest

The author declares that the research was conducted in the absence of any commercial or financial relationships that could be construed as a potential conflict of interest.

## Publisher’s Note

All claims expressed in this article are solely those of the authors and do not necessarily represent those of their affiliated organizations, or those of the publisher, the editors and the reviewers. Any product that may be evaluated in this article, or claim that may be made by its manufacturer, is not guaranteed or endorsed by the publisher.
